# Compliance Rate of Surgical Antimicrobial Prophylaxis and its Association with Knowledge of Guidelines Among Surgical Residents in a Tertiary Care Public Hospital of a Developing Country

**DOI:** 10.7759/cureus.4776

**Published:** 2019-05-29

**Authors:** Muhammad Zubair Satti, Muhammad Hamza, Zaina Sajid, Omaima Asif, Hassaan Ahmed, Syed Muhammad Jawad Zaidi, Umer Irshad

**Affiliations:** 1 Psychiatry, Rawalpindi Medical University and Allied Hospitals, Rawalpindi, PAK; 2 Psychiatry, Rawalpindi Medical University, Rawalpindi, PAK; 3 Surgery, Rawalpindi Medical University, Rawalpindi, PAK; 4 Miscellaneous, Rawalpindi Medical University, Rawalpindi, PAK; 5 General Surgery, Rawalpindi Medical University, Rawalpindi, PAK

**Keywords:** surgical antimicrobial prophylaxis, compliance rate

## Abstract

Introduction

Surgical antimicrobial prophylaxis (SAP) means the administration of antibiotics in surgical practice, and it reduces the likelihood of surgical site infections (SSIs). Inappropriate SAP practice regarding the prescription, timing, and duration of antibiotic use prolongs the hospital stay of patients, increases patient morbidity (by exposing them to the adverse effects of antibiotics), promotes bacterial resistance, and puts an economic burden on health care.

While developed countries regularly monitor and revise their SAP protocols, there are only a few such researches in developing countries, which is a major setback to proper surgical care.

Objectives of the study

This study aims to compare the practice of SAP in a tertiary health care hospital of a developing country Pakistan, with internationally recommended protocols and evaluate the impact of knowledge of international guidelines on SAP practice. The results of the study will highlight important shortcomings in prophylactic practice in the hospital and help develop recommendations to improve SAP practice and ensure better surgical care for patients.

Materials and methods

An observational, cross-sectional study was conducted in the general surgery unit of Holy Family Hospital (HFH), Rawalpindi, Pakistan, from March 2017 to November 2017 during which antimicrobial prophylaxis of 150 general surgery procedures was documented on the basis of six international SAP criteria, which were “indication for use of prophylaxis, timing of preoperative dose, choice of drug, route of administration, duration of postoperative prophylaxis, and the assessment of beta-lactam allergy.” The compliance rate (number of procedures following all the six criteria) was calculated for each operating surgical resident.

A questionnaire was formulated that assessed the knowledge of 33 surgical residents working at that time regarding the above- mentioned six variables of SAP by six close-ended questions. Their responses were then compared to their compliance rate by chi-square analysis and binary logistic regression in SPSS version 23 (IBM Corp, Armonk, NY, US). A p-value of less than or equal to 0.05 was considered significant.

The required ethical approval was obtained from the departmental heads as well as institutional research forum.

Results

Seventy-four of 150 observed procedures followed all the six international criteria of SAP, giving a compliance rate of 49.33%. Seventeen out of 33 (51%) surgical residents were aware of the guidelines. A chi-square analysis revealed a highly significant association between the awareness of guidelines and the number of compliant procedures performed by a resident (p<0.000). Forty-five out of 74 compliant procedures were performed by residents who were aware of the guidelines (61% of compliant procedures). The odds ratio for awareness and correct prophylaxis was 4.064 (p<0.000).

Conclusions

The study indicates an overall low compliance rate of 49.33% regarding surgical antimicrobial prophylaxis (SAP) practice in a public health care hospital of a developing country. The most common cause of non-compliance was prolonged postoperative prophylaxis. This study also shows that the knowledge of international guidelines significantly improves the prophylaxis practice by about four times. Hence, proper SAP compliance rate can be increased by actively educating and monitoring surgical residents.

## Introduction

Surgical site infections (SSIs) are defined as infections occurring at the incision site or deep tissue space within 30 days after surgery [1]. They are among the most common nosocomial infections around the world, causing pain and discomfort to surgical patients [2]. SSIs are involved in one-third of postoperative mortalities and are responsible for 8% of all deaths due to nosocomial infections [2]. SSIs are also an established, yet preventable, cause of prolonged hospital stays and a major economic burden on health care [3]. Despite the improvements in preventive measures, the rate of surgical infections is feared to increase because of the advent of more complex and prolonged surgeries and a growing number of elderlies, diabetics, immunosuppressed, and cancer patients, all of which are risk factors for SSIs [4-5]. In developing countries like Pakistan, with limited health care and lack of adequate research in the matter, surgical site infections pose an even greater challenge, as implied by a recent global study that shows that the overall rate of SSI increases from 7.4% in high-income countries to 20% in low-income countries [2].

Although many factors contribute towards SSIs, contamination of the surgical site by pathogenic microbes remains the most important [6]. Surgical antimicrobial prophylaxis (SAP) is defined as the administration of antibiotics in surgical practice, and it reduces the likelihood of surgical site infections by preventing the growth of such pathogens [1]. Due to proper antimicrobial prophylaxis, the incidence of SSIs has been reduced by 50% in some procedures and proper SAP is now considered the most important preventive measure against SSIs [7]. However, the literature suggests inappropriate SAP practice regarding the prescription, timing, and duration of antibiotic use worldwide, which prolongs the hospital stay of patients, increases patient morbidity (by exposing them to the adverse effects of antibiotics), promotes bacterial resistance, and puts an economic burden on health care [5,8-9]. Thus, it is the need of the hour to practice a rational SAP and minimize the side effects mentioned above.

Proper SAP practice requires good knowledge of international guidelines among the hospital staff and regular evaluations of prophylaxis protocols. While the developed countries regularly monitor and revise their SAP protocols, there is a paucity of such researches in developing countries, which is a major setback to proper surgical care. This study compares the practice of surgical antimicrobial prophylaxis (SAP) in a tertiary health care public hospital of a developing country, Pakistan, with internationally recommended protocols and elaborates the impact of the knowledge of international guidelines on SAP practice. The findings of this study will highlight important shortcomings in prophylactic practice in the hospital and help develop recommendations to improve SAP practice and ensure better surgical care for the patients.

## Materials and methods

Study design

A descriptive, cross-sectional study was conducted in the general surgery unit of Holy Family Hospital, Rawalpindi, Pakistan, from March 2017 to November 2017 during which antimicrobial prophylaxis of 223 elective general surgery procedures was observed. Patients with immunodeficiency, cancer, concurrent infections, and those undergoing contaminated surgical procedures were excluded from the study. Pediatrics and gynecological surgeries and procedures with a primary indication for prophylaxis (cardiac, valvular, and orthognathic) were also excluded from the study.

Of the 223 procedures observed, 150 met the criteria and were further documented. Data regarding SAP in each procedure were collected by the investigators and subsequently entered on a standard data collection form.

Criteria for comparison

Six common variables of surgical prophylaxis were observed and compared with international guidelines. These were “indication for use of prophylaxis, the timing of the pre-operative dose, choice of drug, route of administration, duration of postoperative prophylaxis and the assessment of beta-lactam allergy.” Overall compliance was calculated based upon criteria used in previous studies [10-11], and only those procedures were labeled “compliant” in which all the six variables were individually compliant with the guidelines. A procedure in which any one or more of the six variables were not practiced according to the guidelines was labeled non-compliant.

Questionnaire formation

A questionnaire was formulated that assessed the knowledge of the surgical residents regarding the above-mentioned six variables of antimicrobial prophylaxis by six close-ended questions. Evidenced-based SAP guidelines developed by World Health Organization (WHO) [1], National Institute for Health and Care Excellence (NICE) [9], and Stanford Health Care (SHC) [12] were used as a reference in our study.

Questionnaires (sample shown in the Appendix) were distributed among general surgery residents of the hospital by convenient sampling. Postgraduate trainees (PGTs) in their second to fourth years of surgical residency were included in the study, as they performed antimicrobial prophylaxis in the surgical unit.

Grouping of surgical residents

Residents were divided into two groups based on their response to the questions. The criteria for assessing compliance (mentioned above) was also used to assess the awareness of surgical residents and only those residents who correctly answered all the questions were labeled “aware of guidelines.” One or more incorrect answers by the residents marked them as “not being aware of guidelines.”

Statistical techniques

The descriptive analysis was performed using SPSS v23.0 (IBM Corp, Armonk, NY, US) to characterize the population parameters and study variables.

Chi-squared analysis was then performed between the resident’s awareness of international guidelines and practice of antimicrobial prophylaxis. A p-value of less than or equal to 0.05 was considered statistically significant. Binary logistic regression and odds ratio were also calculated for compliant procedures based on the awareness of guidelines.

## Results

Population parameters and the characteristics of procedures studied are provided in Table 1.

**Table 1:** Characteristics of Population and Procedures Studied n=number

Characteristics of Population Studied	Characteristics of Procedures studied			
	Clean contaminated procedures (n=115)		Clean procedures (n=35)	
	Laparoscopic cholecystectomy	59	Inguinal hernia repair	12
Total procedures observed=223	Open Cholecystectomy	25	Breast surgery	7
Procedures included in study=150	Appendectomy	20	Umbilical hernia repair	7
Male patients in study=81	Hepatobiliary procedure	7	Incisional hernia repair	5
Female patients in study=69	Small intestine procedure	4	Thyroidectomy	4
Mean age of population=39.3 years	The compliance rate was 56.21%		The compliance rate was 28.57%	

The individual compliance rate of six variables is shown in Table 2.

**Table 2:** Compliance Rate of Six Variables of Prophylaxis n=number

Criteria of prophylaxis (SAP)	Indication according to international guidelines	Residents aware of the guideline (n=33)	Practice observed in the procedures (n=150)	Compliance rate (%)	% of non-adherence to guidelines
Indication for prophylaxis	Indicated only in 125 of 150 cases of study	29	Given in all 150 cases	83.33%	16.67%
Timing of pre-operative dose	Give within 120 minutes before the procedure	33	Given within 120 minutes in all 150 procedures.	100%	__
Choice of drug	1^st^ choice for all procedures is Cefazolin	19	Cefazolin administered in only 86 procedures	57.33%	42.67%
Route of administration	Use Intra-venous (IV) route	33	Intravenous (IV) route used in all 150 procedures	100%	__
Duration of postoperative prophylaxis	Limit the duration to less than 24 hours	18	The duration was less than 24 hours in only 79 of 150 procedures	52.67%	47.33%
Assessment of beta-lactam allergy	Test for beta-lactam allergy in all patients	20	Beta-lactam allergy was assessed in only 92 cases	61.33%	38.67%
Number of compliant procedures was 74 (49.33%)					

The results of the questionnaire-based study are indicated in Table 3 along with the compliance rate of the two groups of surgical residents. A chi-squared analysis revealed a highly significant association between the awareness of guidelines and the number of compliant procedures performed by a resident (p<0.000). Forty-five out of 74 compliant procedures (63.38%) were performed by residents aware of the guidelines.

**Table 3:** Groups of Surgical Residents and Their Compliance Rates n=number

Group of surgical residents	Number and Percentage	Awareness of guidelines	Procedures performed (n=150)	Compliant procedures (n=74)	Non-compliant procedures (n=76)	Compliance (%)
GROUP A RESIDENTS	17 (51.51%)	Aware of international guidelines	66	45	21	68.18%
GROUP B RESIDENTS	16 (48.48%)	Not aware of international guidelines	84	29	55	34.52%
Chi-square shows a significant association between compliant procedures and awareness (p< 0.000)						

Table 4 shows the classification table of Block 1 of binary logistic regression, indicating that our model correctly predicts 66.7% cases as compared to 50.7% cases of Block 0. The odds ratio was 4.064 for the significance of (p<0.000).

**Table 4:** Binary Logistic Regression: Classification Table Block 1

OBSERVED		PREDICTED		
		Were they correct		Percentage correct
		no	yes	
Were they compliant	no	55	21	74.4
	yes	29	45	60.8
Overall percentage				66.7
The Odds ratio for compliance based on awareness was 4.064 (p< 0.000).				

## Discussion

Implementation of a proper surgical antimicrobial prophylaxis (SAP) is a challenge worldwide [13], as it requires good knowledge of international recommendations and repeated evaluations of prophylaxis practice.

In our study, prophylaxis was used in all 150 procedures, whereas it was indicated only in 125 of 150 cases (83.33% compliant) if NICE guidelines are adhered to, which state that uncomplicated clean surgeries should be performed without prophylaxis because large clinical trials have shown no benefit of the use of antibiotics in these procedures [9]. Of the procedures, 16.67% (25 out of 150) in our study were non-complaint regarding indication for use of prophylaxis. The finding of antibiotic use in all routine surgeries is consistent with another study conducted in Pakistan, which reports antibiotic use in 99% observed surgeries [14]. Studies in other developing countries also report non-compliance regarding the indication of prophylaxis. An Ethiopian study showed 19.6% procedures received antibiotics without indications [15]. The administration of prophylaxis in clean surgeries is deemed unnecessary and contraindicated by international guidelines because such unnecessary antibiotic use is the most important factor responsible for the development of antimicrobial resistance (AMR), a global health crisis as it complicates the treatment of bacterial infections and leads to the eventual loss of efficacy of that particular drug [16]. AMR is widespread in developing countries due to the lack of routine checks on antibiotic use, over-the-counter availability of many broad-spectrum antibiotics, and inappropriate antibiotic prescription in a surgical setting [17]. Moreover, this practice also puts an extra economic burden on health care, a system which already receives a very low proportion of the budget in developing countries.

The problem of antimicrobial resistance is compounded by another observation in our study. According to Stanford Health Care (SHC) guidelines, the recommended prophylactic drug in all procedures (except in patients with a serious beta-lactam allergy) is a first-generation cephalosporin, cefazolin [12]. The goal is to administer the drug with a moderate spectrum of activity, targeting only the suspected surgical pathogens so that the development of antimicrobial resistance is prevented [9], and cefazolin fits this criterion. In our study, 64 procedures (42.67%) used broad-spectrum third-generation cephalosporin ceftriaxone for prophylaxis instead of cefazolin. This finding is consistent with a previous study conducted across Pakistan, which reported the use of ceftriaxone in 57.6% procedures [18] and another study reported that 60.7% of procedures received ceftriaxone [19]. A study in India also reported the use of third-generation cephalosporins for routine prophylaxis [20] while an Italian study showed a compliance rate of only 9.8% regarding the choice of drug in the hospital [21]. A study based in Nekemte Hospital reported ceftriaxone use in 84% procedures [15]. These findings indicate a general trend among the doctors in developing countries to prefer broad-spectrum drugs in the surgical setting, which aggravates the above-mentioned problem of antimicrobial resistance. With the routine use of broad-spectrum drugs, multidrug-resistant (MDR) bacteria are eventually selected, which cause serious postoperative complications [16]. Thus, our study points out two factors, the widespread use of antibiotics and preference for broad-spectrum drugs, in a surgical setting that promote antibiotic resistance. The emergence of resistant bacteria has complicated the treatment of many infections such as pneumonia and tuberculosis, increasing the length of hospital stays, medical costs, and mortality rates of the affected patients [17]. Fearing the loss of efficacy of antibiotics, the World Health Organization (WHO) recommends a change in the practice regarding antibiotic use by doctors, prescribing them only when necessary [14]. This recommendation needs to be transformed into surgical practice to prevent the emergence of resistant pathogens and their spread to the community.

Regarding the timing of preoperative prophylaxis, the guidelines state that the optimal time for the first dose of the prophylactic drug is within 120 minutes before incision [1]. It is important to follow the timing because administering antibiotics before 120 minutes of incision increases the risk of surgical site infections, as the concentration of the drug may fall below the adequate levels needed for its effects [1]. All 150 patients in our study received preoperative prophylaxis in this time frame, giving a 100% compliance rate to this guideline. A previous study in Pakistan showed that 80% of procedures were compliant regarding the timing of the preoperative dose and 18% of these procedures received the drug just before incision [22]. A study conducted in India indicated that the timing of preoperative dose was not according to any guidelines and, in some cases, started six hours before surgery [20], and a study in Italy also indicated variable adherence with this guideline [21]. The practice of administering the preoperative dose inside the operation theater (OT) by the anesthesiologist in charge ensured full compliance with this guideline in our study.

International guidelines recommend the intravenous (IV) administration of prophylactic drugs to ensure steady and predictable drug levels [1]. The practice of surgical residents in our study followed this guideline, giving a 100% compliance rate regarding the route of administration of the prophylactic drug. The finding is consistent with a similar study in the Philippines, which also reports 100% compliance with this guideline [23].

In 71 of 150 (47.33% non-compliant) observed procedures, postoperative prophylaxis was continued for more than 24 hours after surgery based on the wrong assumption that such practice may decrease the infection risk even further, giving a compliance rate of 52.67% to this guideline. A previous study in Pakistan reported only 26.1% adherence to this guideline [22] and a study in Saudi Arabia indicated 58.2% compliance regarding the duration of postoperative prophylaxis [23]. The guidelines state, with strong evidence, that in all surgeries (except cardiac, vascular, and orthognathic procedures), prolonging the prophylaxis for more than 24 hours after the surgery has no additional benefit [1]. Rather, it has been associated with a prolonged hospital stay of patients, increased patient morbidity due to the risk of the side effects of antibiotics, development, and spread of bacterial resistance [1] and as a risk factor for Clostridium difficile infection, the cause of pseudomembranous colitis [24]. In the current era, much research is focused on reducing patient recovery time after surgery and a unanimous Enhanced Recovery After Surgery (ERAS) protocol has been developed to optimize surgical care. A key aspect of ERAS is to minimize fluid administration in surgical patients [25]. The development of diarrhea either due to Clostridium difficile infection or direct gastrointestinal distress caused by prolonged antibiotic use requires the use of intravenous (IV) fluids and contradicts ERAS protocols, increasing the post-operative hospital stay of patients. This also puts an unnecessary physical, emotional, and economic burden on the patient and consumes hospital resources and manpower by attending to such patients. Thus, the developing countries need to follow the international guidelines regarding postoperative prophylaxis to minimize the patient’s exposure to antibiotics and reduce the average length of hospital stay of surgical patients.

According to the literature, up to 35% percent of surgical patients suffer from beta-lactam allergy, and it may lead to perioperative anaphylaxis when severe [26], which makes it necessary to assess the patients’ beta-lactam allergy status before administering the prophylactic cephalosporins. An immunoglobulin E (IgE)-mediated allergically reaction exhibited by the patient during the test, contraindicates cephalosporins, and alternative non-beta-lactam drugs, such as vancomycin or clindamycin, are then administered [26]. Although cephalosporins were administered in all procedures in our study, a beta-lactam allergy was assessed only in 92 procedures (61.33% compliant). In the remaining 58 procedures (38.67% non-compliant), cephalosporins were administered in the operation theater without knowing the allergy status. A case of anaphylaxis in a patient undergoing cholecystectomy was observed in the study due to such practice. The patient was administered ceftriaxone on the operating table just before the incision and developed hypotension and decreasing oxygen saturation during the surgery, requiring epinephrine administration to prevent a fatal outcome. This highlights the importance of knowing the beta-lactam allergy status of surgical patients before administering cephalosporins. Studies in other developing countries do not include this variable in assessing the prophylactic practice but a rational SAP practice must tend to the prevention of any drug-related adverse effects that may occur during the surgery, which makes the assessment of beta-lactam allergy status necessary.

Our study indicates an overall low compliance ratio of 49.33% when all six criteria of antimicrobial prophylaxis practice are considered. In other words, of 150 procedures in which SAP was analyzed, only 74 were compliant with international guidelines regarding all the six variables and 76 deviated from the guidelines in one or more of these criteria. The compliance rate was higher in clean-contaminated surgeries (56.21%) than in clean surgeries (28.57%). This observation contradicts with a similar study conducted in Qatar in which the compliance rate of clean procedures (66%) was higher than clean-contaminated procedures (34%) [6]. The markedly low compliance rate in clean surgeries observed in our study was due to the use of antibiotics in all observed surgeries, whereas uncomplicated clean surgeries do not require prophylaxis according to the international guidelines.

Other related studies in the literature also report the failure of compliance with international guidelines, establishing it as a global health care problem. Table 5 shows a comparison of the compliance rate in our study with those of similar studies around the world:

**Table 5:** Comparison of Compliance Rate with Other Studies

Country of study	Overall compliance rate	The compliance rate of individual criteria of prophylaxis					
		Indication	Timing	Chosen drug	Duration of post-operative dose	Route of drug administration	Assessment of beta-lactam allergy
Qatar [6]	46.5%	___	___	68.5%	40.7%	100%	__
India [18]	Low compliance Rate	___	No guidelines followed	3^rd^ generation cephalosporins used frequently	__	100%	__
Philippines [21]	13%	___	45%	44%	67%	100%	__
Italy [19]	40%	72.3%	46.6%	40%	40.7%	100%	__
Saudi Arabia [22]	___	48%	91%	3.6%	58.2%	100%	__
Pakistan [20]	Less than 50%	Prophylaxis used in 99% routine surgeries	___	3^rd^ generation cephalosporins in more than 50 % cases	26.1%	100%	__
Present study	49.33%	83.33%	100%	57.33%	52.67%	100%	61.33%

While the previous studies only reported the shortcomings in SAP practice, our research also focuses on one of the factors responsible for the low compliance rate. It was proposed that awareness of guidelines regarding SAP among surgical residents may have a direct bearing upon the prophylaxis practice. A chi-squared analysis revealed that the number of compliant procedures performed by surgical residents is significantly associated with their awareness of international guidelines (p<0.000). A binary logistic regression between the independent categorical variable (awareness of guidelines) and the dependent nominal variable (whether the procedures were compliant or not) in the two groups of surgical residents. The results were statistically significant and the proposed model correctly classified 66.7% of cases, an improvement over 50.7% of cases classified by the baseline model. This validates our proposed model that the awareness of guidelines increases the number of compliant procedures performed by the resident.

The odds ratio calculated for a confidence interval of 95% was 4.064 (with lower and upper limits of 2.047 and 8.070, respectively) and (p<0.000). This means that a surgical resident who is aware of the international guidelines on SAP is, on average, four times more likely to perform correct prophylaxis and yield a 100% compliance rate than a resident who is not aware of the guidelines.

Other studies also argued that lack of awareness regarding the guidelines contributes to inappropriate SAP practice [6] but our study provides statistical evidence of the importance of awareness of international guidelines in administering the correct prophylaxis. This result is significant because it indicates that a proper SAP practice can be achieved by imparting proper knowledge regarding SAP guidelines among the current and future surgical residents. Adherence to evidence-based practice will decrease the rate of surgical site infections, provide better surgical care to patients, and reduce the cost burden on the health care budget in developing countries.

To ensure the practice of rational prophylaxis, the authors recommend increasing the awareness of international guidelines of antimicrobial prophylaxis among doctors and surgical residents by arranging workshops and teaching courses. Also, repeated evaluations of prophylactic practice must be conducted to identify the factors responsible for low compliance and develop strategies to counter them. A unanimous national prophylaxis protocol should be developed to standardize SAP practice in hospitals.

Limitations

Our study has certain limitations. First, due to the lack of national guidelines, SAP practice may differ among surgical residents and different compliance rates may be obtained if a different study population is selected. Second, the observation that surgeons with knowledge of guidelines also perform inappropriate SAP in some cases (21 such cases in our study) should be further evaluated and the factors for this finding should be identified and tackled. Third, the study only concerns a tertiary care hospital in Rawalpindi and more extensive multicenter studies should be conducted to elaborate on the results.

## Conclusions

The study indicates an overall low compliance rate of 49.33% regarding surgical antimicrobial prophylaxis (SAP) practice in a tertiary health care hospital of Rawalpindi, which predisposes the patients to the unnecessary side effects associated with a non-compliant SAP. The compliance rate was higher in clean-contaminated surgeries than in clean surgeries and the most common cause of non-compliance was post-operative prophylaxis of more than 24 hours. Forty-five of 74 compliant procedures were performed by residents aware of the guidelines. The study also shows that proper awareness of international guidelines regarding SAP increases the likelihood of proper prophylaxis by four times. Interventions are needed to ensure rational antimicrobial prophylaxis practice by implementing the above-mentioned recommendations so that better surgical care is provided to the patients and the hazards of inappropriate SAP are avoided.

## Appendices

The proper prophylaxis

Below are some questions regarding Surgical Antimicrobial Prophylaxis (SAP) in your hospital. Please take out some of your precious time and answer them to the best of your knowledge.

Name of Resident: ______________

Q1: In your opinion, is SAP needed in all routine surgeries?

a)       Yes

b)      No, some surgeries can be performed without SAP

Q2: What is the optimal time for administering prophylactic drug?

a)       A day before surgery

b)      3 hours before surgery

c)       Within 120 minutes of surgery

d)      4 hours before surgery

Q3: Which is the preferred drug for routine prophylaxis?

a)       Cefazolin

b)      Ceftriaxone

c)       Any other drug (please mention _____________)

Q4: What is the preferred route for administering the prophylactic drug?

a)       IV route

b)      Oral route to minimize side effects

c)       IM injection

Q5: What is your routine duration for postoperative prophylaxis?

a)       Less than 24 hours after surgery

b)      2 days after surgery

c)       3 days after surgery

Q6: In your practice, do you take the patient’s history of beta-lactam allergy before administering prophylactic drugs?

a)       Yes

b)      No

AWARENESS STATUS: ___________________

## References

[1] Leaper DJ, Edmiston CE (2017). World Health Organization: global guidelines for the prevention of surgical site infection. J Hosp Infect.

[2] Collaborative G (2017). Determining the worldwide epidemiology of surgical site infections after gastrointestinal resection surgery: protocol for a multicentre, international, prospective cohort study. BMJ Open.

[3] Badia JM, Casey AL, Petrosillo N, Hudson PM, Mitchell SA, Crosby C (2017). Impact of surgical site infection on healthcare costs and patient outcomes: a systematic review in six European countries. J Hosp Infect.

[4] Guohua X, Cheng K, Li J, Kong Q, Wang C, Nanyuan Y, Guohua X (2015). Risk factors for surgical site infection in a teaching hospital: a prospective study of 1,138 patients. Patient Prefer Adherence.

[5] Hagel S, Scheuerlein H (2014). Perioperative antibiotic prophylaxis and antimicrobial therapy of intra-abdominal infections. Visc Med.

[6] Abdel-Aziz A, El-Menyar A, Al-Thani H (2013). Adherence of surgeons to antimicrobial prophylaxis guidelines in a tertiary general hospital in a rapidly developing country. Adv Pharmacol Sci.

[7] Duclos G, Zieleskiewicz L, Leone M (2017). Antimicrobial prophylaxis is critical for preventing surgical site infection. J Thorac Dis.

[8] Santana RS, Viana A de C, Santiago J da S, Menezes MS, Lobo IMF, Marcellini PS (2014). The cost of excessive postoperative use of antimicrobials: the context of a public hospital. Rev Col Bras Cir.

[9] (2019). NICE. Surgical site infection. https://www.nice.org.uk/guidance/qs49/resources/surgical-site-infection-pdf-2098675107781.

[10] Nabor MIP, Buckley BS, Lapitan MCM (2015). Compliance with international guidelines on antibiotic prophylaxis for elective surgeries at a tertiary-level hospital in the Philippines. Healthc Infect.

[11] Sami OA, IbrahimMahadi S, Ahmed MD, ElMakki Ahmed M (2015). Antibiotic prophylaxis in clean and clean-contaminated surgery and surgical site infection in Khartoum Teaching Hospital. Sudan Med J.

[12] (2019). Stanford Health Care. SHC surgical antimicrobial prophylaxis guidelines. http://med.stanford.edu/bugsanddrugs/guidebook/_jcr_content/main/panel_builder_584648957/panel_0/download/file.res/SHC_SurgProphylaxisGuidelines.pdf.

[13] Sullivan E, Gupta A, Cook CH (2017). Cost and consequences of surgical site infections: a call to arms. Surg Infect.

[14] Alemkere G (2018). Antibiotic usage in surgical prophylaxis: a prospective observational study in the surgical ward of Nekemte Referral Hospital. PLoS One.

[15] Tanwar J, Das S, Fatima Z, Hameed S (2014). Multidrug resistance: an emerging crisis. Interdiscip Perspect Infect Dis.

[16] Ayukekbong JA, Ntemgwa M, Atabe AN (2017). The threat of antimicrobial resistance in developing countries: causes and control strategies. Antimicrob Resist Infect Control.

[17] Allegranzi B, Bischoff P, de Jonge S (2016). New WHO recommendations on preoperative measures for surgical site infection prevention: an evidence-based global perspective. Lancet Infect Dis.

[18] Malik AZ, Ali Q (2015). Surgical site infections after elective surgery in Pakistan: SURGIPAK study. J Rawalpindi Med Coll.

[19] Kaur R, Salman MT, Gupta NK, Gupta U, Ahmad A, Verma VK (2015). Presurgical antibiotic prophylaxis pattern in an Indian tertiary care teaching hospital. JK Sci.

[20] Quattrocchi A, Barchitta M, Maugeri A (2018). Appropriateness of perioperative antibiotic prophylaxis in two Italian hospitals: a pilot study [Article in Italian]. Ann Ig.

[21] (2019). WHO. Antimicrobial resistance. https://www.who.int/antimicrobial-resistance/en/.

[22] Rahman R, Arshad A (2015). Assessment of surgical prophylaxis in Pakistani patients undergoing abdominal or pelvic surgeries: the results of the NASPAK Registry. Pak J Surg.

[23] Ahmed NJ, Jalil MA, Al-shdefat RI, Tumah HN (2015). The practice of preoperative antibiotic prophylaxis and the adherence to guideline in Riyadh hospitals. Bull Env Pharmacol Life Sc.

[24] Balch A, Wendelboe AM, Vesely SK, Bratzler DW (2017). Antibiotic prophylaxis for surgical site infections as a risk factor for infection with Clostridium difficile. PLoS One.

[25] Melnyk M, Casey RG, Black P, Koupparis AJ (2011). Enhanced recovery after surgery (ERAS) protocols: time to change practice?. Can Urol Assoc J.

[26] Hermanides J, Lemkes BA, Prins JM, Hollmann MW, Terreehorst I (2018). Presumed β-lactam allergy and cross-reactivity in the operating theater: a practical approach. Anesthesiology.

